# Targeted Gene Mutations in the Forest Pathogen *Dothistroma septosporum* Using CRISPR/Cas9

**DOI:** 10.3390/plants11081016

**Published:** 2022-04-08

**Authors:** Hannah M. McCarthy, Mariana Tarallo, Carl H. Mesarich, Rebecca L. McDougal, Rosie E. Bradshaw

**Affiliations:** 1 BioProtection Aotearoa, School of Natural Sciences, Massey University, Palmerston North 4472, New Zealand; m.tarallo@massey.ac.nz (M.T.); r.e.bradshaw@massey.ac.nz (R.E.B.); 2 BioProtection Aotearoa, School of Agriculture and Environment, Massey University, Palmerston North 4472, New Zealand; c.mesarich@massey.ac.nz; 3 Scion, New Zealand Forest Research Institute Ltd., Rotorua 3010, New Zealand; rebecca.mcdougal@scionresearch.com

**Keywords:** Dothistroma needle blight, pine pathogen, Dothideomycete, gene disruption, fungal transformation, CRISPR/Cas9, *Dothistroma*, DSB repair, homologous recombination

## Abstract

Dothistroma needle blight, caused by *Dothistroma septosporum*, has increased in incidence and severity over the last few decades and is now one of the most important global diseases of pines. Disease resistance breeding could be accelerated by knowledge of pathogen virulence factors and their host targets. However, this is hindered due to inefficient targeted gene disruption in *D. septosporum*, which is required for virulence gene characterisation. Here we report the first successful application of CRISPR/Cas9 gene editing to a Dothideomycete forest pathogen, *D. septosporum.* Disruption of the dothistromin pathway regulator gene *AflR*, with a known phenotype, was performed using nonhomologous end-joining repair with an efficiency of >90%. Transformants with a range of disruption mutations in *AflR* were produced. Disruption of *Ds74283*, a *D. septosporum* gene encoding a secreted cell death elicitor, was also achieved using CRISPR/Cas9, by using a specific donor DNA repair template to aid selection where the phenotype was unknown. In this case, 100% of screened transformants were identified as disruptants. In establishing CRISPR/Cas9 as a tool for gene editing in *D. septosporum*, our research could fast track the functional characterisation of candidate virulence factors in *D. septosporum* and helps set the foundation for development of this technology in other forest pathogens.

## 1. Introduction

The ability to specifically mutate genes of interest is of utmost importance to our understanding of how plant pathogens cause disease, and it is based on the careful analysis of interaction phenotypes observed between pathogen mutants and their plant hosts. The pathogen–host interaction database (PHI-base) exemplifies this approach by providing an inventory of curated pathogen gene mutations that affect disease phenotypes [1]. The increased availability of genome sequences and associated in planta transcriptome data facilitates the identification of candidate genes from plant pathogens that can be tested for roles in virulence, pathogenicity, or other types of interactions. However, the application of molecular tools such as targeted gene deletion or editing in forest pathology is almost nonexistent compared to their use in agricultural and horticultural settings [2,3].

Some Dothideomycete fungi are pathogens of forest trees, and among these are *Dothistroma septosporum* and its sister species *Dothistroma pini*, which cause Dothistroma needle blight (DNB) of pines and some other Pinaceae species [4]. DNB is globally one of the most important pine diseases, and increases in incidence and severity are associated with changes in the climate [4,5]. Learning more about how these pathogens interact with their hosts could help in breeding or selection for disease resistance, and in developing new tools for disease management [3,6].

Genomic and transcriptomic resources for *D. septosporum* [7,8] enabled the identification of candidate virulence genes [6]. Targeted gene mutation is regularly performed in *D. septosporum* using the traditional method of homologous recombination based on protoplast transformation, with the aim of determining gene function. For example, genes involved in the biosynthesis of the anthraquinone toxin dothistromin were deleted by this method and showed dothistromin to be a virulence factor in DNB [9]. Other deleted *D. septosporum* genes include potential virulence and avirulence genes [3,10], and those involved in gene regulation [11,12]. However, in *D. septosporum*, the homologous recombination method is inefficient, with only a small proportion of transformants being gene knockout mutants. The development of CRISPR/Cas9 technology for targeted gene modification and its success in filamentous fungi [13,14] led to our CRISPR/Cas9 trial in *D. septosporum*.

Clustered regularly interspaced short palindromic repeats (CRISPR) is part of an adaptive immune system in prokaryotes that protects against invasion from phages and plasmids [15,16]. CRISPR operates through two main components: Cas nuclease proteins, which cut the DNA to produce a double-stranded break (DSB), and CRISPR RNAs (crRNAs), which direct the Cas protein [17] to the protospacer adjacent motif (PAM) site [18]. In prokaryotes, the genetic memory of previous viral invasions can be stored as CRISPR spacers and used to enable crRNAs to defend against subsequent invasions [16,18]. For the use of CRISPR/Cas9 as a gene editing technology, crRNA is adapted into a single-guide RNA (sgRNA) [19] and designed to match to an existing PAM site in the target gene, allowing for specific gene editing [19,20].

Since the first report of CRISPR/Cas9 gene editing in filamentous fungi [13], this technology has been used in many different fungal species [21], including plant pathogens [2,22]. Pathogenic Dothideomycete species where CRISPR/Cas9 technology was successfully applied include *Alternaria alternata* [23], *Leptosphaeria maculans* [24], *Parastagonospora nodorum* [25]. and *Venturia inaequalis* [26]. In *P. nodorum*, CRISPR/Cas9 gene editing technology was recently used to characterise the *SnTox5* gene, showing that it encodes a virulence factor responsible for inducing programmed cell death and facilitating mesophyll colonisation in wheat [27].

Here, we set out to establish the CRISPR/Cas9 gene editing system in *D. septosporum* using synthetic sgRNA to obtain *AflR* gene mutants (Joint Genome Institute (JGI) ID: Ds75566). *AflR* is the pathway regulator gene for the biosynthesis of the dothistromin virulence factor [28]. Mutants with a disrupted *AflR* gene have a dothistromin-deficient phenotype, based on an earlier study that involved gene replacement using traditional homologous recombination methods [28]. We also trialled the use of sgRNA with a donor DNA for CRISPR/Cas9-mediated homology-dependent repair to obtain mutants in a different gene whose mutant phenotype was unknown.

## 2. Results

### 2.1. Transformation of D. septosporum with CRISPR/Cas9 and sgRNAs Targeting AflR Yielded a High Percentage of Dothistromin-Deficient Transformants

**Figure 1:**
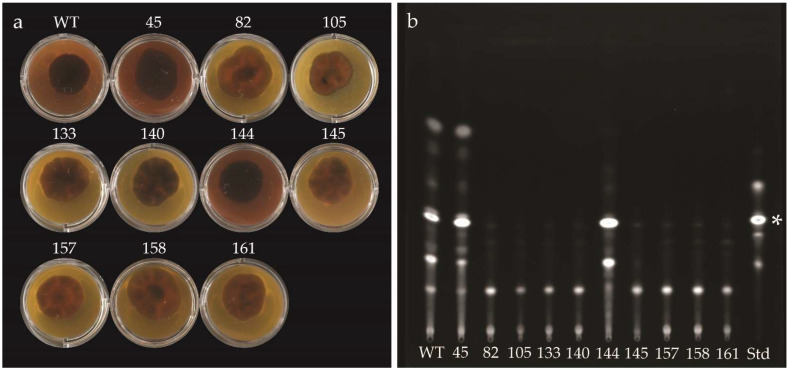
Presence or absence of dothistromin from wild-type (WT) *Dothistroma septosporum* and CRISPR/Cas9 *AflR* transformants selected for investigation. (**a**) Visual analysis of dothistromin production when grown on Dothistroma medium (DM) supplemented with 100 µg/mL CuSO_4_.5H_2_O (known to enhance dothistromin production; Figure S2). Presence of dothistromin shown by red-brown pigmentation of the media, as seen in DM supporting growth of WT fungus, and transformants 45 and 144. Transformants were photographed 4 weeks postinoculation. (**b**) Thin-layer chromatography (TLC) analysing dothistromin production in *AflR* transformants and WT fungus. Position of dothistromin is shown in standard (Std), highlighted with a white asterisk. Dothistromin was visible in samples representing WT fungus, and transformants 45 and 144.

*D. septosporum* was transformed using two different sgRNAs to increase the chance of obtaining an *AflR* gene mutant. After two rounds of subculturing on hygromycin B selective media, 1 transformant was obtained from sgRNA AflR1, and 47 transformants from sgRNA AflR2. From a preliminary phenotypic screen, 45 of these transformants (including the one from sgRNA AflR1) were suggested to be *AflR* knockouts because they had a dothistromin-deficient phenotype, as indicated by the absence of red-brown pigmentation in the surrounding media on which they were cultured. The three other transformants had a dothistromin-producing phenotype similar to the wild-type (WT) fungus. Representative samples are shown in Figure 1a.

As a starting point to verify which transformants were *AflR* mutants, and to identify which types of mutations were caused by CRISPR/Cas9 gene editing, PCR screening was performed using primers HM89 and HM90 that bind either side of *AflR* (Figure S1). Only four transformants gave a PCR product that was the same size as the WT fungus (1.6 kb); three of these had a dothistromin-producing phenotype in a visual screen (37, 45, and 144), while transformant 140 was dothistromin-deficient. Two transformants (145 and 158) gave larger PCR products than those of the WT fungus, while all other screened transformants either did not have a PCR product or had several nonspecific PCR products. Together, these results suggested that over 90% of the CRISPR/Cas9 transformants had a mutation in the *AflR* gene that led to the dothistromin-deficient phenotype.

### 2.2. A Single-Nucleotide Deletion and Insertions Ranging from 387 bp to 2.8 kb Were Identified among AflR CRISPR/Cas9 Transformants

The different types of mutations caused by CRISPR/Cas9 gene editing were investigated in a subset of ten transformants (all from sgRNA AflR2). Subset selection was based on transformants that gave a specific PCR product of WT size or larger, and a representative selection of transformants that displayed different patterns of nonspecific PCR amplification. Dothistromin phenotypes and thin-layer chromatography (TLC) profiles of these ten transformants showing the presence or absence of dothistromin are shown in Figure 1.

**Figure 2:**
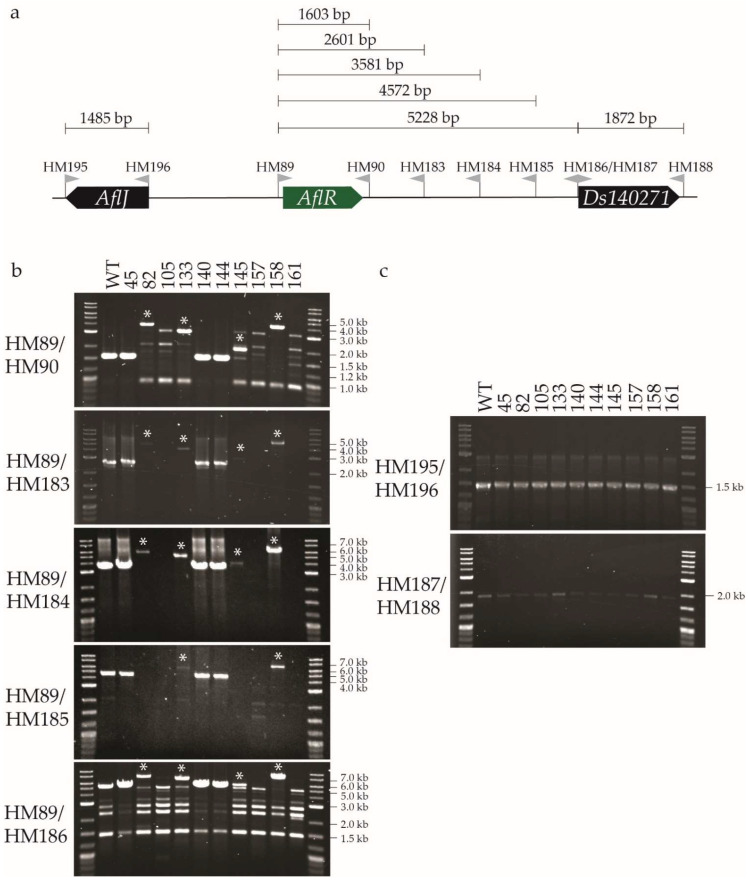
PCR screening to characterise *Dothistroma septosporum AflR* CRISPR/Cas9 transformants. (**a**) Primers used to screen the *AflR* region. Green bar is *AflR*, and black bars are flanking genes, labelled with either name or JGI protein ID. Positions of primers are illustrated by grey flags with the primer name above. Distances between the primer pairs shown above the primers. (**b**) PCR screen of *AflR* gene and intergenic region between *AflR* and *Ds140271*. (**c**) PCR screen of genes upstream and downstream of *AflR*. Name of each transformant screened shown at the top of each group of gel pictures. PCR products larger than wild-type (WT)-sized PCR products labelled with a white asterisk. Relevant labels of the DNA ladder shown to the right of each gel picture, and primers used in each PCR shown on the left.

**Figure 3:**
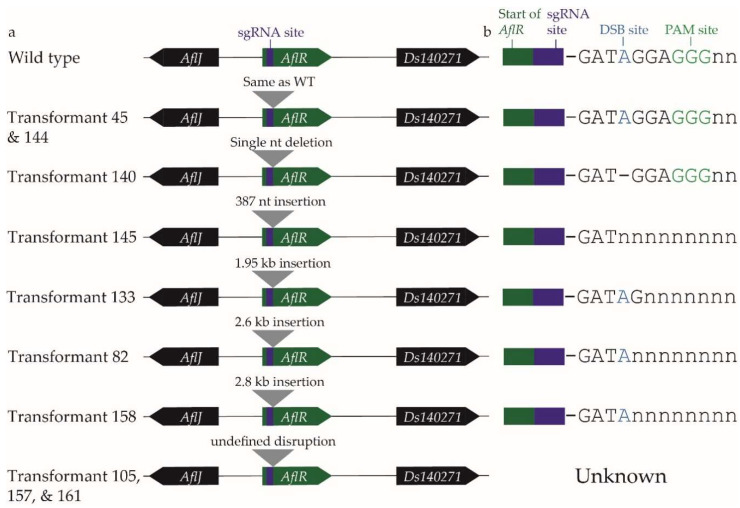
Summary of mutations identified from *Dothistroma septosporum AflR* CRISPR/Cas9 transformants. (**a**) Visual representation of *AflR* locus including flanking genes in each transformant. Purple bar in *AflR* is the CRISPR/Cas9 sgRNA AflR2 binding site. Mutations, if present, indicated by a grey triangle and labelled with type of mutation identified through phenotype, PCR analysis, and PCR amplicon sequencing. (**b**) Specific locations within sgRNA AflR2 binding site where CRISPR/Cas9 disruptions were observed. sgRNA AflR2 binding site is 23 bp; end with double-strand break (DSB) site (in blue) and protospacer adjacent motif (PAM) site (in green) shown as nucleotides, and the rest illustrated by a purple bar. Where a transformant had an insertion, the insertion start position is shown through truncation of the sgRNA AflR2 binding site sequence. Single-nucleotide deletion in transformant 140 shown by a dash in the sequence. For transformants 105, 157, and 161, no PCR amplification was achieved in the *AflR* region; therefore, no sequencing was performed (labelled “undefined/unknown”).

PCR analysis was then carried out using genomic DNA from the ten transformants (Figure 2), and selected PCR products were sequenced (Figure 3). Southern blot analysis with two restriction enzymes was also performed to confirm the results (Figure S3). As in the preliminary screen, PCR with primers HM89/HM90 gave a 1.6 kb product with genomic DNA from the WT fungus and transformants 45, 140, and 144 (Figure 2b). Sequencing of these PCR products confirmed that transformants 45 and 144 had the WT *AflR* sequence and are thereby not *AflR* disruptants, as suggested from their dothistromin-producing phenotype and TLC profile (Figure 1). Transformant 140 did not appear to produce dothistromin (Figure 1) despite the WT-sized PCR product, and sequencing identified a single adenine nucleotide deletion at the double-strand break (DSB) site, resulting in a frame-shift mutation (Figure 3b). These results were further supported by WT-sized fragments obtained from transformants 45, 140, and 144 in Southern hybridisations (Figure S3).

A PCR product larger than that from the WT fungus (1.6 kb) was amplified from transformants 82 (4.2 kb), 133 (3.5 kb), 145 (2 kb), and 158 (4.4 kb), consistent with the preliminary PCR screen (Figure 2b). Insertions of various sizes ranging from 387 nt to 2.8 kb were estimated from the *AflR* PCR (HM89/HM90) product sizes, with additional evidence from the full-sequence analysis of smaller PCR products (Figure 3). The site of insertion in these transformants was at the DSB site or up to 2 nt downstream towards the protospacer adjacent motif (PAM) site (Figure 3b). Southern hybridisation analysis of genomic DNA digested with *Hin*dIII or *Xho*I showed that each of these four insertion transformants (82, 133, 145, 158) lacked a band corresponding to the WT *AflR* gene, confirming their status as *AflR* mutants (Figure S3). Transformants 82, 133, and 158 had the expected *Xho*I fragments, but smaller *Hin*dIII hybridising bands than those expected, indicative of extra *Hin*dIII sites in the inserted DNA. Transformant 145 was unusual in showing larger hybridising bands for both *Hin*dIII and *Xho*I digests (Figure S3) despite being the transformant with the smallest insertion of 387 bp. These results are unusual and not supported by previous PCR analysis, suggesting that whole-genome sequencing is required to determine the full extent of CRISPR/Cas9 editing in this transformant.

Genomic DNA from transformants 105, 157, and 161 did not give a specific PCR product (Figure 2b), consistent with the preliminary PCR screen. It is possible that these transformants contain a large indel that could not be captured by the PCR screen using primers HM89 and HM90. To investigate this, primers were designed to amplify genes on either side of *AflR*: *AflJ* and *Ds140271*. As expected, the WT fungus and all transformants for which a PCR product was obtained from the *AflR* screen showed amplification of these two neighbouring genes. The genomic DNA of transformants 105, 157, and 161 also amplified *AflJ* and *Ds140271* (Figure 2c). This suggested that, if these transformants each contained a large indel, it did not disrupt the genes located either side of *AflR*.

To identify the hypothesised indel in these three transformants (105, 157, 161), primers were designed at ~1 kb intervals between *AflR* and *Ds140271* (Figure 2a). PCR amplification of the set of 10 transformants using the HM89 forward primer with each of the nested reverse primers HM183, HM184, HM185, and HM186 (Figure 2b) gave PCR products consistent with those expected on the basis of PCR results with the *AflR* gene HM89/HM90 primers. In some cases, PCR products were very faint or nonspecific bands were observed, the latter occurring in all samples for the widely spaced HM89/HM186 primer pair (the largest distance apart in this primer series). Interestingly, for putative large-indel transformants 105, 157, and 161, no PCRs involving the region spanning between *AflR* and *Ds140270* gave a specific product. Southern hybridisation analysis of transformants 105, 157, and 161 confirmed the disruption of the WT *AflR* locus as expected. It also revealed differently sized fragments among the three transformants for each restriction enzyme (Figure S3), suggesting that these transformants were the products of independent insertion events with differently sized inserts and additional restriction enzyme recognition sites. It was not possible to estimate the insert sizes on the basis of available information. In summary, these results show that a variety of disruptions in *AflR* were produced from CRISPR/Cas9 gene editing in *D. septosporum*.

### 2.3. Insertions in Dothistroma septosporum AflR Transformants Match Components of the CRISPR/Cas9 Plasmid

**Figure 4:**
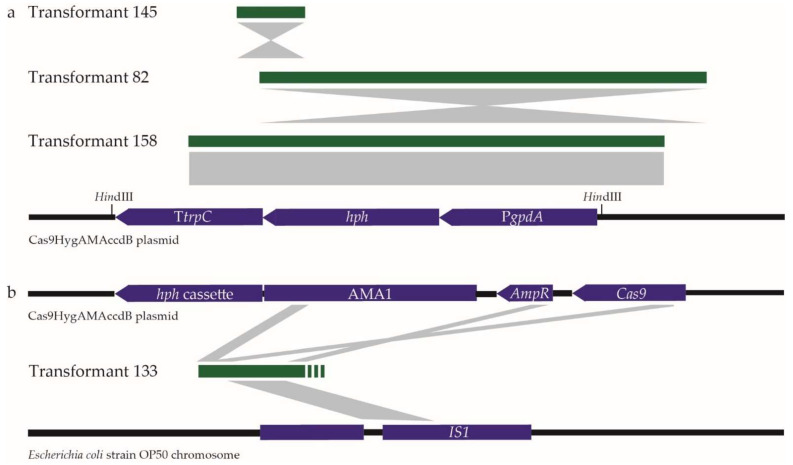
Insertions in *Dothistroma septosporum* CRISPR/Cas9 *AflR* transformants and matching sequences. (**a**) Insertions in transformants 145, 82, and 158 that match part of the *hph* (hygromycin B resistance gene) cassette from the CRISPR/Cas9 plasmid (Cas9HygAMAccdB). (**b**) Part of insertion in transformant 133 that matches different regions of the CRISPR/Cas9 plasmid and a region of an *Escherichia coli* strain OP50 chromosome. Insertions were identified by PCR analysis and PCR amplicon sequencing. Insertions indicated by green bars align to matching regions of the Cas9HygAMAccdB plasmid or *E. coli* chromosome. Grey rectangles or triangles underneath and above insertions indicate relative orientation compared to the *AflR* gene. *Hin*dIII restriction enzymes sites near the *hph* cassette are labelled. Black lines indicate part of the Cas9HygAMAccdB plasmid sequence or *E. coli* chromosome, and labelled purple bars indicate genes or plasmid components (T*trpC* terminator, *hph* cassette, P*gpdA* promoter, AMA1 autonomously replicating sequence, *AmpR β-lactamase* (ampicillin resistance) gene, *Cas9* gene). PCRs performed to support expected insertion sizes and content of transformants 82 and 158 shown in Figure S4.

Transformants with insertions that gave a larger product than that from the WT fungus in the *AflR* PCR (primers HM89/HM90) were analysed by PCR amplicon sequencing. The 387 bp insertion from transformant 145 was identified to match part of the CRISPR/Cas9 plasmid (Cas9HygAMAccdB) used in transformation, specifically the C-terminal region of the hygromycin B resistance gene (*hph*) and the *trpC* terminator of the *hph* cassette (Figure 4). Partial sequencing of the larger insertions in transformants 82 and 158 also showed matches to the *hph* cassette (Figure 4). PCR product size and sequence information suggested that the insertion in transformant 82 included the *gpdA* promoter and the full *hph* coding sequence, while the insertion in transformant 158 appeared to include the entire *hph* cassette but in the opposite orientation to the two other transformants (Figure 4). Transformants 82 and 158 thus both contained regions of the *hph* cassette that include a *Hin*dIII recognition site, accounting for the additional *Hin*dIII site predicted from the Southern hybridisation results (Figure S3). Transformant 133 contained an insertion that matched different parts of the CRISPR/Cas9 plasmid, including regions of the AMA1 autonomously replicating sequence, and parts of the *Cas9* and ampicillin resistance (*AmpR*) genes, interspersed with a segment of DNA that was not from the plasmid but matched bacterial insertion (*IS1*) sequences, including those from *Escherichia coli*. Together, these results showed that, in several transformants, partial copies of the plasmid used for CRISPR/Cas9 editing in *D. septosporum* were inserted at the DSB.

### 2.4. Donor DNA Enables Homologous Recombination in CRISPR/Cas9 Mutation of a Putative Virulence Gene of Unknown Phenotype

Ds74283 is a small secreted protein from *D. septosporum* that triggers plant cell death in a nonhost species *Nicotiana benthamiana* and in the host species *Pinus radiata* (Figure S5), and is a putative virulence gene [6]. *Ds74283* expression is strongly upregulated at the transcriptional level during mid and late in planta stages that mark the start and end of the necrotrophic stage of pine infection, when compared with the early (biotrophic) stage and growth in culture [8].

**Figure 5:**
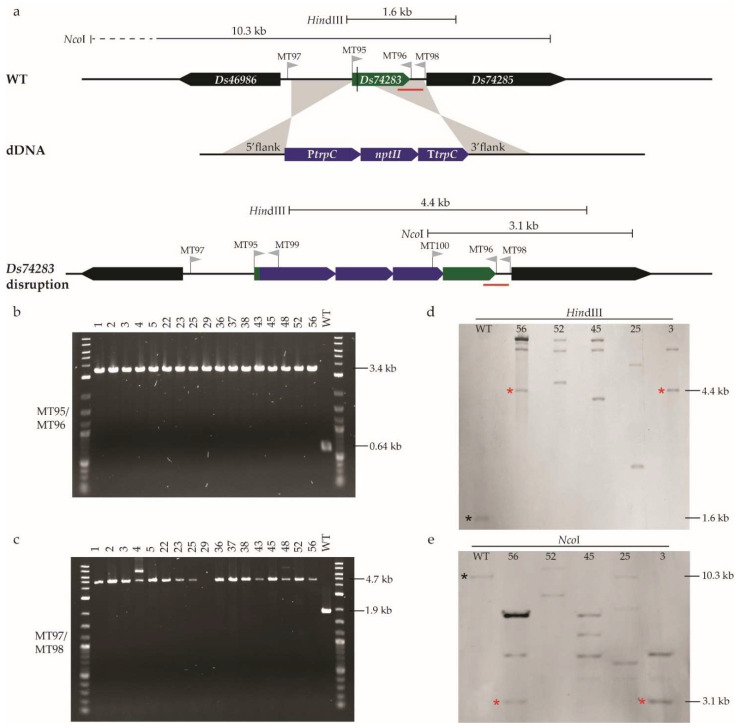
*Dothistroma septosporum* CRISPR/Cas9 *Ds74283* disruption and confirmation by PCR and Southern hybridisation. (**a**) Schematic diagram showing disruption of *Ds74283* and insertion of *nptII* geneticin gene cassette (P*trpC*-*nptII*-T*trpC*) through homologous recombination using donor DNA (dDNA) as template. dDNA constructed with two flanks (5′ and 3′) from *Ds74283*, starting 3 bp from double-strand break (shown as black vertical line crossing the gene), with *nptII* cassette in the middle. Positions of primers illustrated by grey flags with primer name above. Restriction enzyme sites and probe binding site used for Southern hybridisation, and fragment sizes expected from disruption of *Ds74283* by insertion of the *nptII* cassette. Red line shows probe binding site. (**b**) PCR with primers MT95, which binds to the start codon of *Ds74283*, and MT96, which binds to the stop codon of the gene. *Ds74283* mutants should have a product of 3.4 kb, while wild-type (WT) fungus should have one of 0.64 kb. (**c**) PCR with primers MT97 and MT98, which bind either side of the target genomic region. *Ds74283* mutants should have a product of 4.7 kb, and the WT fungus 1.9 kb. Relevant labels of the DNA ladder are shown on the right of each gel. (**d**,**e**) Southern hybridisation of digests ((**d**) *Hin*dIII; (**e**) *Nco*I) from *D. septosporum* WT fungus and five candidate *Ds74283* mutants, 3, 25, 45, 52 and 56, using a digoxigenin (DIG)-11-dUTP-labeled probe binding to the 3′ flank region of *Ds74283* that was present in the dDNA. Expected fragment sizes marked with red asterisk for *Ds74283* mutants and black asterisk for WT fungus. Each of the five *Ds74283* mutants sampled from independent transformation plates.

Unlike *AflR*, the mutant phenotype of *Ds74283* was unknown, so a screen for targeted mutants based on phenotype was not possible. To maximise the chance of obtaining mutants, we transformed *D. septosporum* with plasmids containing donor DNA (dDNA) in addition to sgRNA that targets *Ds74283*. Using CRISPR/Cas9 alone with sgRNA requires the fungus to repair the DSB using nonhomologous end joining (NHEJ), which is an efficient but error-prone DNA repair pathway [29]. The other DNA repair system, homologous recombination, occurs in the presence of a template, generating a faithful copy of the template DNA. Therefore, an exogenous repair template, in this case, dDNA consisting of a resistance selection marker (*nptII*, which confers resistance to geneticin) flanked by *Ds74283* sequences and designed to disrupt the gene, was delivered into the cell to allow for homologous recombination repair to occur (Figure 5a).

The co-transformation of *D. septosporum* with sgRNA and dDNA targeting *Ds74283* produced 82 transformants, of which 42 were stable on selective hygromycin B and geneticin media. Of these, 17 were randomly selected to be single-spore purified in the presence of geneticin and maintained on nonselective medium. All 17 transformants showed the same visual phenotype as that of the WT fungus and were screened by PCR with two sets of primers that each spanned the *Ds74283* gene (MT95/MT96 and MT97/MT98; Figure 5a). Compared to the WT fungus, all 17 *D. septosporum* transformants had PCR products that were 2.8 kb larger in size, as expected for gene disruption by homologous recombination (Figure 5b,c). Five transformants from five independent transformation plates were also analysed by PCR across the junctions of the flanking sequences and the inserted *nptII* geneticin resistance cassette (using primers MT97/MT99 and MT100/MT98) and results were consistent with disruption of *Ds74283* by integration of the *nptII* gene (Figure S6).

The same set of five transformants (3, 25, 45, 52 and 56) were selected for verification by Southern hybridisation (Figure 5d,e). As expected, none of them showed hybridisation to fragment sizes corresponding to the WT fungus, suggesting the successful disruption of *Ds74283*. However, only two of the transformants (3 and 56) showed hybridisation to fragment sizes expected from integration of the *nptII* gene by homologous recombination at the disruption site. Moreover, all five transformants showed additional hybridising fragments that did not correlate with the predicted sizes from partial digests or insertion of whole dDNA plasmids. In summary, in all screened transformants, *Ds74283* was disrupted, and Southern hybridisation analysis showed that, in two of them, it was possible to detect the expected disruption size band, although additional hybridising bands were present.

## 3. Discussion

This study showed that targeted disruption of the *D. septosporum* dothistromin regulator gene *AflR* and a gene encoding small secreted protein Ds74283 could be achieved using CRISPR/Cas9 technology. To the best of our knowledge, this is the first application of this technology in a Dothideomycete forest pathogen, and only the second in any forest pathogen, with a prior report of CRISPR/Cas9 editing being performed in the Dutch elm disease pathogen *Ophiostoma novo-ulmi* [30]. In *D. septosporum*, we report a high efficiency (>90%) of *AflR* gene disruption using CRISPR/Cas9 without a donor repair template, and 100% disruption of *Ds74283* with a repair template in screened transformants. This finding greatly facilitates the functional analysis of genes identified from genomic and transcriptomic studies [8], as achieved with Dothideomycete pathogens *Lasiodiplodia theobromae* [31] and *Leptosphaeria maculans* [32], to characterise new virulence factors.

CRISPR/Cas9 transformation of *D. septosporum* to disrupt *AflR* was performed using two different sgRNAs. Despite having similar on-target scores, and the transformation being performed with the same conditions in the same experiment, significantly different numbers of colonies were obtained. While variability in sgRNA activity has been reported [26], our transformation strategy selected for the uptake of the autonomously replicating CRISPR/Cas plasmid rather than sgRNA activity. Therefore, the difference in the number of transformants obtained when using each sgRNA was likely due to differences in plasmid uptake and persistence.

The disruption of *AflR* was highly efficient using CRISPR/Cas9 with NHEJ repair; however, a range of transformant types was produced. Of the studied transformants, one had a single-nucleotide deletion, four had insertions ranging from 387 bp to 2.8 kb, and three had undefined disruptions. The gene editing of *V. inaequalis* using the same CRISPR/Cas9 transformation method also produced a variety of different mutant types, but had lower gene disruption efficiency of 16.7% [26].

Several *D. septosporum AflR* transformants contain insertions that matched part of the CRISPR/Cas9 plasmid used in transformation, specifically all or part of the hygromycin B resistance gene *hph* despite the CRISPR/Cas9 plasmid containing a fungal autonomously replicating sequence [26]. The insertion of a CRISPR plasmid into fungal transformants was first reported by Fuller et al. [33], who found the insertion of partial and complete copies of the transforming plasmid, resulting in larger insertions than those reported in this study. Subsequent studies consistently found the insertion of the complete plasmid at the DSB site [34] or the insertion of large plasmid fragments [35,36]. Fuller et al. [33] suggested that the insertion of the CRISPR plasmid or of partially degraded parts of the plasmid could occur at the DSB site through the NHEJ mechanism. More recent research suggested that the integration of CRISPR plasmids can occur following their cleavage with nucleases that are present in protoplast lysates during transformation, which allows for incorporation by the NHEJ mechanism, essentially using the cleaved plasmid product as a dDNA molecule [35]. All these studies that reported the insertion of partial or complete copies of a CRISPR plasmid [33,34,35,36] used protoplast transformation and hygromycin B selection. In our study, *AflR* transformants were cultured four times on hygromycin B selective media, providing strong selection for the stable integration of the *hph* gene. To reduce such integration events, it is preferable to subculture on a nonselective medium after the first round of selection on transformation plates.

In CRISPR/Cas9 homology-directed repair, DSB repair is based on complementarity with a dDNA template, in most cases resulting in a perfect repair of the DNA. Therefore, an exogenous repair template with the desired alteration, such as the insertion of an antibiotic resistance selection marker, can be delivered into the cell to allow for homologous recombination repair to replace the endogenous segment. For genes whose phenotype cannot be predicted, the use of dDNA can facilitate faster and more efficient screening for transformants. In this study, we disrupted a candidate virulence gene from *D. septosporum* which codes for a small, secreted cell death elicitor. For *Ds74283* disruption, we co-transformed *D. septosporum* with the same autonomously replicating plasmid containing the *Cas9* endonuclease gene as used for *AflR*, along with the specific sgRNA and a dDNA plasmid harboring a selective resistance marker, with flanking arms of approximately 1 kb. We found successful disruption of *Ds74283* in all stable transformants tested.

The strategy that we used for *Ds74283* CRISPR/Cas9 disruption was successfully used in different fungal and oomycete species. In *Phytophthora sojae*, homologous recombination was stimulated using the *nptII* gene in between 1 kb flanking sequences from the candidate gene, as used in our study [14]. The combination of a Cas9 protein and dDNA-sgRNA expression plasmid resulted in almost 90% of *Colletotrichum sansevieriae* transformants with the candidate gene disrupted [37], with flanking sequences (1–1.5 kb) resulting in efficient dDNA recombination. However, shorter flanking arms can also work efficiently; 100% efficiency of homologous integration of the dDNA in CRISPR/Cas9 transformation of *Beauveria bassiana* was obtained when 250 bp flanking sequences were used [38].

For *D. septosporum* CRISPR/Cas9 transformation with dDNA, PCR analysis indicated very high frequency of homologous recombination, with all transformants having the correctly sized band corresponding to the integration of the *nptII* cassette. Southern blot analysis, however, revealed that even though *Ds74283* was disrupted in all transformants tested, all of them had extra hybridising fragments. None of those fragments matched in size with the integration of an extra copy of the selective marker cassette, or partial digestion of genomic DNA. These extra fragments might be due to partial integration of parts of the dDNA in other regions of the genome which are therefore also being targeted by the hybridisation probe. Nevertheless, we successfully disrupted *Ds74283* and detected the expected size of disruption band in two independent transformants.

CRISPR/Cas9 disruption of *D. septosporum* was very efficient using both the NHEJ and dDNA homologous recombination repair mechanisms. If the phenotype of a mutant is known, and the aim is simple disruption of the gene, we recommend using the NHEJ repair system. If the phenotype is not known, using the dDNA-mediated homologous recombination repair is the best option. However, in both systems, we observed unusual effects from CRISPR/Cas9 disruption, including the insertion of the CRISPR plasmid, and additional fragments. Reducing the time spent growing transformants on selective media would reduce the selective pressure for integration of DNA containing selectable marker genes and most likely lead to cleaner disruption mutants. In experiments where the effects of CRISPR/Cas9 mutation must be fully understood, whole-genome sequencing may be needed to determine if there are off-target mutations.

To the best of our knowledge, our research is the first to report successful and efficient CRISPR/Cas9 transformation in a Dothideomycete forest pathogen. This work enables investigation of the role of the *Ds74283* cell death elicitor by determining the virulence of *Ds74283* mutants on pine. More broadly, CRISPR/Cas9 technology also accelerates functional studies of other putative virulence candidates in *D. septosporum*‒pine interactions [6]. This enables us to address complex questions such as what triggers the transition from biotrophy to necrotrophy in this hemibiotrophic pathogen [8].

CRISPR/Cas9 technology has immense potential in the development of new methods of plant disease control in general, but is greatly underutilised in forest pathosystems largely due to the complexities of tree genomes and the long life cycles of trees [2]. However, disease control can be improved by the application of CRISPR/Cas9 to forest pathogens and the trees themselves [2]. CRISPR/Cas9 provides an efficient tool for functional analysis of potential disease-related pathogen genes that can be used in turn to identify plant host targets and decipher the complexities of plant‒pathogen interactions [2,14]. Furthermore, pathogen genes that are found to have virulence functions, thanks to functional analysis by CRISPR/Cas9 mutagenesis, could also be deployed for developing new methods of disease control, for example by serving as targets for RNA-induced gene silencing, potentially restricting pathogen growth [39,40]. We hope the success we report in *D. septosporum* encourages the use of CRISPR/Cas9 technology in other forest pathogens and contribute to the global fight for forest health.

## 4. Materials and Methods

### 4.1. Culturing of Dothistroma septosporum

WT *D. septosporum* strain NZE10 was used in this study. WT fungus and CRISPR/Cas9 transformants were routinely cultured on Dothistroma medium (DM) [41] at 22 °C for 7 days. Culturing was performed by grinding mycelium in sterile water and spreading it over the plate. To facilitate visual phenotype screening for dothistromin production in *AflR* transformants, the DM medium was supplemented with 100 µg/mL CuSO_4_.5H_2_O to elevate dothistromin production (Figure 1 and Figure S2). For single-spore purification of *AflR* CRISPR/Cas9 transformants, sterile water was added to a sporulating plate for 10 min, then spores collected by pipetting and streaked onto a new plate. Two rounds of spore purification were performed. For the purification of *Ds74283* CRISPR/Cas9 transformants, ground mycelium was used instead of spores (two rounds performed). Mycelium for gDNA extraction was harvested by either culturing onto a DM plate with cellophane and scraping off mycelium when grown or inoculating 25 mL of DM broth in a 125 mL flask with ~200 µL ground mycelium, incubating for 7 days at 22 °C, shaking at 160 rpm, and harvesting the mycelium through a sterile nappy liner.

### 4.2. Construction of CRISPR/Cas9 Plasmid

CRISPR sgRNA selection is an important part of the CRISPR/Cas9 transformation process that can impact the transformation efficiency. Two sgRNAs targeting the *AflR* gene, and one targeting *Ds74283*, were selected from the first exon (Table 1) to maximise the effects of any frameshift mutations that might be caused during DSB repair [42]. sgRNAs were identified using the “find CRISPR sites” tool in Geneious (v9.1.8 software) and selected for the highest on-target activity score and an off-target score of 100% (Table 1). After sgRNAs had been selected, they were produced and inserted into the CRISPR plasmid Cas9HygAMAccdB using previously described methods [26]. Plasmids were screened after insertion of the sgRNA and sequenced with primer M139 and the reverse gRNA primer. All primers used in this study are detailed in Table S1.

### 4.3. Construction of Donor DNA Plasmid for Homologous Recombination

The dDNA (template for homologous recombination) was constructed with two flanks from the gene of interest (*Ds74283*), with the *nptII* cassette (P*trpC*-*nptII*-T*trpC*) in between. The 5′ flank (from nucleotide 714,367 to 715,332 on scaffold 9) and 3′ flank (from nucleotide 715,340 to 716,198 on scaffold 9) both start 3 bp from the DSB (which occurs 3 bp downstream from the PAM site) [19]. The *nptII* cassette was amplified from plasmid pII99 [45], while plasmid pAN7-1 [46], which is a Gibson Assembly-compatible plasmid, was the backbone for dDNA assembly. The 966 bp 5′ and 859 bp 3′ flanks of *Ds74283* were amplified from WT NZE10 *D. septosporum* genomic DNA by PCR. The 2808 bp resistance marker sequence was amplified from plasmid pII99, and the 2591 bp backbone vector sequence was amplified from plasmid pAN7-1. All PCRs were performed with Phusion High-Fidelity DNA polymerase (New England Biolabs, Ipswich, MA, USA). Fragments were then assembled via Gibson Assembly [47], and the correct assembly was verified by sequencing. The resulting dDNA plasmid was linearised with the *Nde*I (New England Biolabs) restriction enzyme prior to fungal transformation.

### 4.4. Generation of Dothistroma septosporum Protoplasts and PEG Transformation

Protoplasts were generated from *D. septosporum* as reported previously [48], except that flasks were initially inoculated with mycelial fragments and incubated at 22 °C for 6–7 days. To digest cell walls, 10 mg/mL Glucanex lysing enzyme (Novozymes, Copenhagen, Denmark) was used, and flasks incubated at 30 °C for 12–16 h with shaking at 100 rpm. Transformation was performed as reported previously [48] with several additions. Protoplasts were diluted to 1.25 × 10^8^ protoplasts/mL in STC buffer [48] then, to 80 µL of protoplast suspension, 20 µL 40% polyethylene glycol (PEG) solution (40% PEG 4000, 50 mM CaCl_2_, 50 mM Tris-HCl, 1M sorbitol) was added. For transformation without a dDNA template, 5 µg Cas9HygAMAccdB plasmid (1 µg/µL) was added, while for transformation with dDNA, 3 µg of Cas9HygAMAccdB plasmid and 5 µg of linearised dDNA were added. The protoplast mixture was vortexed briefly and left on ice for 30 min. Then, 900 µL 40% PEG solution was added, mixed, and left at room temperature (~20 °C) for 20 min. Protoplasts were then plated by mixing 100 µL protoplast solution with 3.5 mL molten (50 °C) regeneration medium (RG: 50 g/L malt extract (Oxoid, Basingstoke, UK), 23 g/L nutrient broth (Oxoid), 0.8 M sucrose, 0.8% bacteriological agar (Pure Science, Wellington, New Zealand)) and overlaying an RG plate (15 mL, 1.5% bacteriological agar). Plates were incubated overnight at 22 °C, then a 5 mL overlay of 0.8% RG was added containing antibiotics for the entire plate (70 µg/mL hygromycin B (Roche, Basel, Switzerland), and 100 µg/mL geneticin (Life Technologies, Carlsbad, CA, USA) if using dDNA). Plates were incubated at 22 °C for at least 2 weeks before colonies appeared. Colonies that grew on the *AflR* transformation plates were transferred to 12-well plates containing DM with 70 µg/mL hygromycin B. Colonies were then subcultured to standard DM plates with hygromycin B and single spore purification was performed. Where dDNA was used, colonies were transferred to 12-well plates containing DM with 70 µg/mL hygromycin B and 100 µg/mL geneticin and then subcultured to DM plates with geneticin for purification using ground mycelium and maintained on nonselective DM.

### 4.5. Screening of Transformants by PCR, Southern Hybridisation, Sequencing and Thin Layer Chromatography

Genomic DNA was initially extracted using a crude method to facilitate screening [49], then later, extractions were performed as described by Doyle and Doyle [50] or by van Kan et al. [51]. Transformants were screened by PCR using gDNA as template and *Taq* DNA polymerase with ThermoPol Buffer (New England Biolabs) following the manufacturer’s protocols. PCRs from which products would be sequenced used Phusion High-Fidelity DNA Polymerase as per the manufacturer’s protocols. PCR amplicon sequencing was performed by the Massey Genome Service (Massey University, Palmerston North, New Zealand).

Confirmation of gene disruption was achieved by Southern hybridisation with a digoxigenin (DIG)-labelled probe using the DIG High Prime DNA Labelling and Detection Starter Kit I (Roche). Approx. 2 µg of genomic DNA from WT *D. septosporum* and transformants of this fungus was digested at 37 °C overnight with the restriction enzymes *Hin*dIII, *Nco*I or *Xho*I (New England Biolabs) and run overnight on a 0.8% agarose gel. After that, DNA was transferred to a positively charged nylon membrane (Roche) and blotted overnight according to Southern et al. [52]. Color detection was performed by enzyme immunoassay using the DIG High Prime DNA Labelling and Detection Starter Kit I (Roche), following manufacturer’s instructions.

The presence of dothistromin was assayed by thin-layer chromatography (TLC) as reported previously [11], except that flasks were inoculated with mycelial fragments, incubated for 7 days, and the TLC was run in toluene:acetone (80:20) solvent acidified with 1% formic acid.

## 5. Conclusions

In this study, we have successfully performed CRISPR/Cas9 gene editing in the Dothideomycete forest pathogen *D. septosporum*. We report efficient gene disruption of >90% when using non-homologous end-joining repair, and 100% in screened transformants that used homologous recombination repair with a donor DNA template. Establishment of CRISPR/Cas9 in *D. septosporum* will greatly facilitate characterisation of effectors from this pathogen, and aid the development of this method in other forest pathogens and Dothideomycete species.

## Figures and Tables

**Table 1:** CRISPR/Cas9 sgRNA protospacers used to target for *Dothistroma septosporum AflR* and *Ds74283* genes.

sgRNA Protospacer	Sequence (5′‒3′) ^2^	Binding Site (bp)	Direction	Off-Target Sites ^3^	Off-Target Score ^4^	On-Target Activity Score ^5^
AflR1	TCACGCGGCTCAGAGTCGAGCGG	Exon 1 (10–32)	Forward	0	100%	0.692
AflR2	ACAAGAAGCAGCAGATAGGAGGG	Exon 1 (267–289)	Forward	0	100%	0.703
Ds74283 ^1^	GTTGTTGTAGGCAGAGACGAAGG	Exon 1 (35–57)	Reverse	0	100%	0.635

^1^ Joint Genome Institute (JGI) ID of *Dothistroma septosporum* protein coding for the candidate gene of interest for disruption. ^2^ Underlined bases are the protospacer adjacent motif (PAM) site. ^3^ Off-targets found in the coding sequence (CDS). ^4^ Off-target score calculated using Geneious (v9.1.8) by scoring against the *D. septosporum* genome, where scores are between 1 and 100 and a high score indicates less off-target activity [43]. ^5^ On-target activity score based on sequence features of the sgRNA, calculated using Geneious (v9.1.8). Score was 0–1, with a score closer to 1 indicating higher activity [44].

## Data Availability

The data presented in this study are available in article and supplementary material.

## Supplementary Materials

The following are available online at https://www.mdpi.com/article/10.3390/plants11081016/s1. Figure S1: PCR screen of *Dothistroma septosporum AflR* CRISPR/Cas9 transformants to amplify full-length *AflR* gene sequence; Figure S2: increased production of dothistromin by *Dothistroma septosporum* in the presence of 100 µg/mL CuSO_4_.5H_2_O; Figure S3: Southern hybridisation analysis of *Dothistroma septosporum AflR* CRISPR/Cas9 transformants; Figure S4: PCR analysis to support estimated insertion size and content of *Dothistroma septosporum AflR* CRISPR/Cas9 transformants 82 and 158; Figure S5: *Dothistroma septosporum* candidate effector Ds74283 induces cell death in *Pinus radiata*; Figure S6: PCR screening to further characterise five *Dothistroma septosporum* CRISPR/Cas9 *Ds74283* transformants. Table S1: primers used in this study.
