# Supplementary material for: Targeted Gene Mutations in the Forest Pathogen Dothistroma septosporum Using CRISPR/Cas9
Source: Plants (Basel). 2022 Apr 8;11(8):1016. doi: 10.3390/plants11081016 (PMC9025729; doi:10.3390/plants11081016)
Supplement: Supplementary file 1 [file plants-11-01016-s001.zip › plants-1633383-supplementary.pdf]

Supplementary Materials

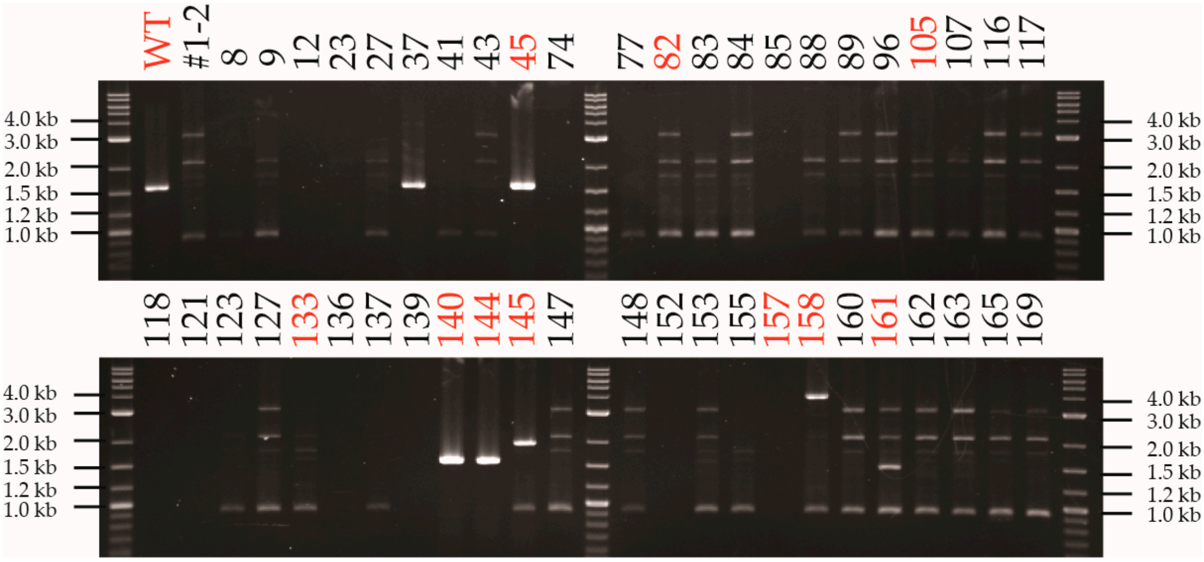

Figure S1. PCR screen of *Dothistroma septosporum AflR* CRISPR/Cas9 transformants to amplify full-length *AflR* gene sequence. Transformant number is at the top of the gel images, with those in red selected for further study. Relevant DNA ladder sizes are shown on the left and right of the gel images. The primers used were HM89 and HM90. All colonies of *D. septosporum* were transformed with sgRNA AflR2, except #1-2, which was transformed with sgRNA AflR1. PCR screening was performed after sub-culturing twice on selective media containing hygromycin B. Nine transformants from sgRNA AflR1 were sub-cultured, but after two rounds of culturing, only one grew. For sgRNA AflR2, when 128 transformants were sub-cultured in the same way, 47 grew. Genomic DNA was extracted using a crude but rapid method [49] to facilitate PCR screening. To check the genomic DNA was of adequate quality for screening by PCR, the *D. septosporum β-tubulin* gene (*Ds68998*) was amplified by PCR prior to this screen with the HM103/HM104 primer pair (result not shown). The correct PCR product was amplified from the genomic DNA of all transformants except transformant 134, which was excluded from the PCR screen shown here. WT: wild-type.

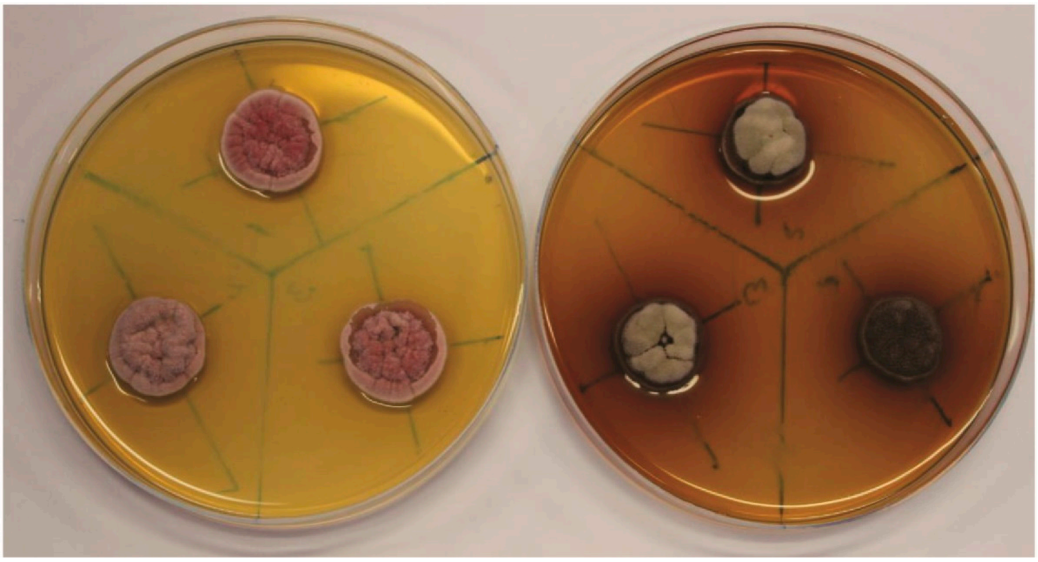

Figure S2. Increased production of dothistromin by *Dothistroma septosporum* in the presence of 100 μg/mL $CuSO_4.5H_2O$. The plate on the left is standard Dothistroma medium (DM), while on the right is DM supplemented with 100 μg/mL $CuSO_4.5H_2O$.

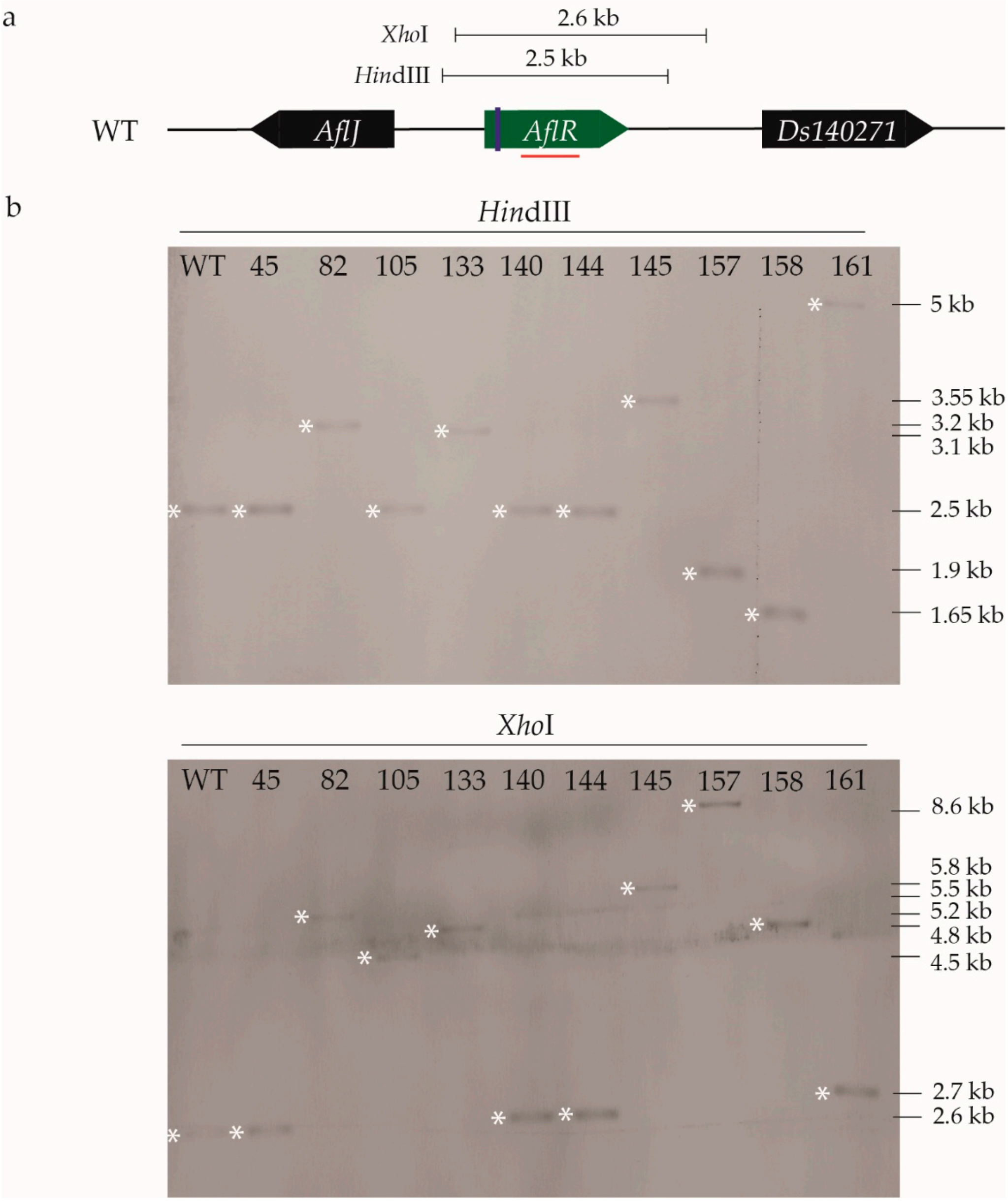

Figure S3. Southern hybridisation analysis of *Dothistroma septosporum AflR* CRISPR/Cas9 transformants. (**a**) Schematic of the wild-type (WT) *AflR* locus and positions of the restriction sites and probe. The green bar is *AflR*, the black bars are flanking genes, the purple line within *AflR* is the sgRNA AflR2 site, and the red line is where the probe binds. Restriction sites and distances between them are shown above. (**b**) Southern hybridization of digested genomic DNA from WT *D. septosporum* and *AflR* CRISPR/Cas9 transformants of this fungus using *Hin*dIII and *Xho*I. A digoxigenin (DIG)-11-dUTP-labelled probe bound to a region of *AflR* downstream of the sgRNA AflR2 site. Hybridizing fragments are marked with an asterisk and sizes are labelled on the right.

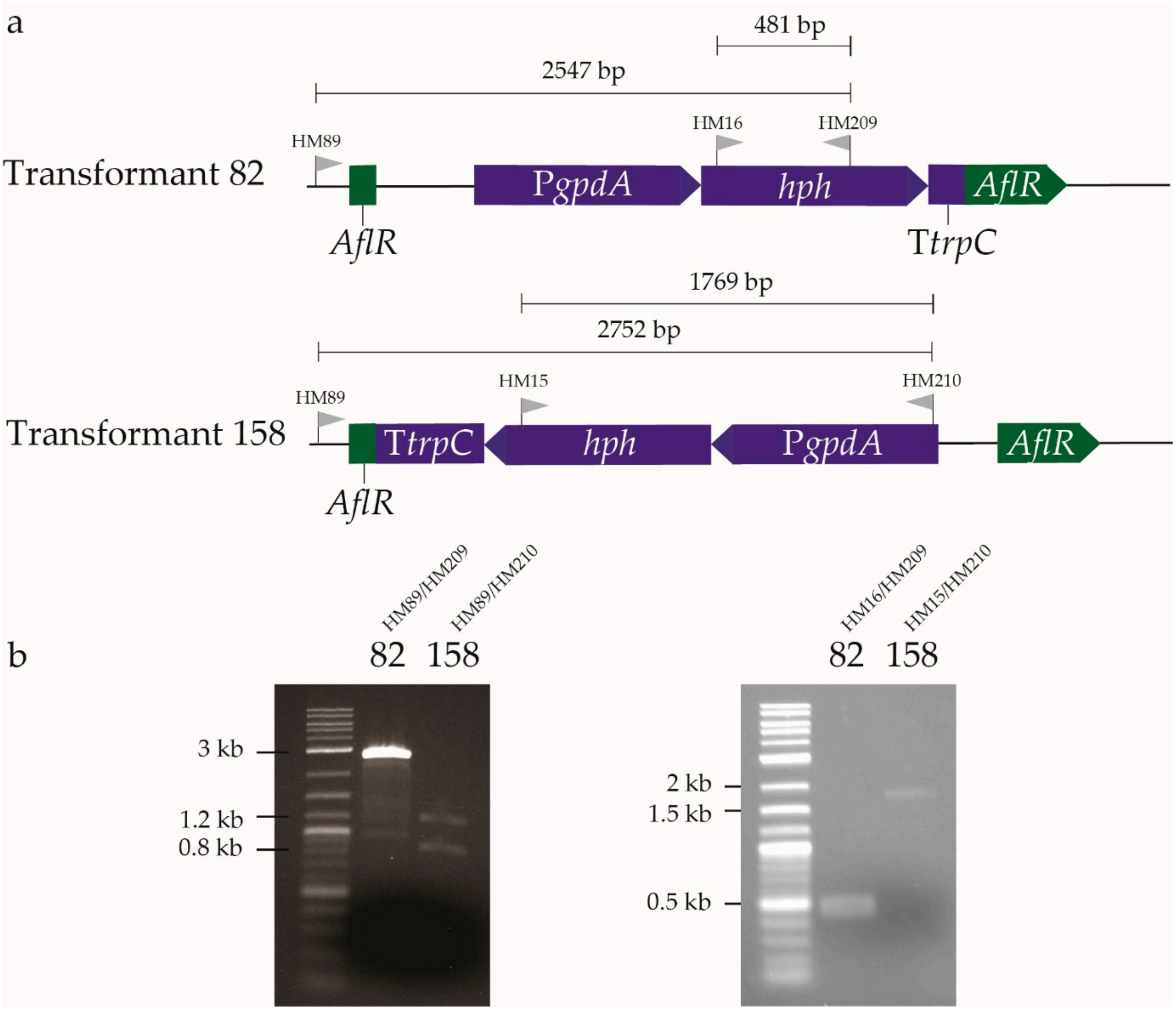

Figure S4. PCR analysis to support estimated insertion size and content of *Dothistroma septosporum AflR* CRISPR/Cas9 transformants 82 and 158. (**a**) Schematic of transformants 82 and 158 with insertions, suggested by previous PCR and PCR amplicon sequencing analysis. *AflR* is the segmented green bar, purple bars are the *hph* cassette, and the black bar between the separated *AflR* regions is part of the CRISPR/Cas9 plasmid (*Cas9HygAMAccd*B). Primer positions are illustrated by grey flags, with the distance between primers shown above. (**b**) PCRs performed on genomic DNA from transformants 82 and 158 using primers detailed in (a). All PCRs amplified the expected band except from genomic DNA of transformant 158 with primers HM89 and HM210 - this was likely due to a shorter insertion than estimated from the PCR product size in earlier screens, leading to lack of a primer binding site and non-specific PCR amplification in transformant 158. Transformant number and primer pairs used are shown at the top of the gel, and relevant labels of the DNA ladder sizes are shown on the left.

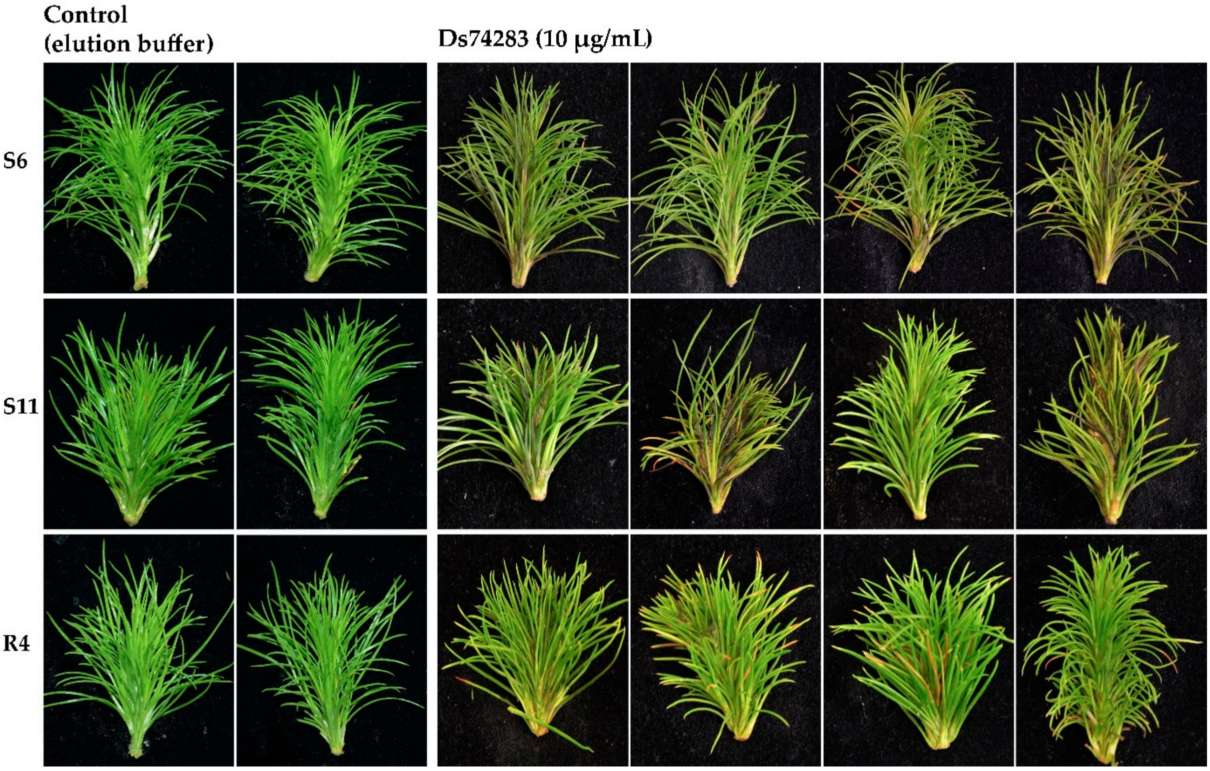

Figure S5. *Dothistroma septosporum* candidate effector Ds74283 induces cell death in *Pinus radiata*. Needles of *P. radiata* genotypes from families that are susceptible (S6 and S11) or tolerant (R4) to *D. septosporum* infection were vacuum-infiltrated with elution buffer (negative control) and purified Ds74283 protein produced by heterologous expression in *Pichia pastoris*, according to Hunziker et al. [6]. Representative photos (from 18 to 24 pine shoots for each treatment) were taken 7 days post-infiltration.

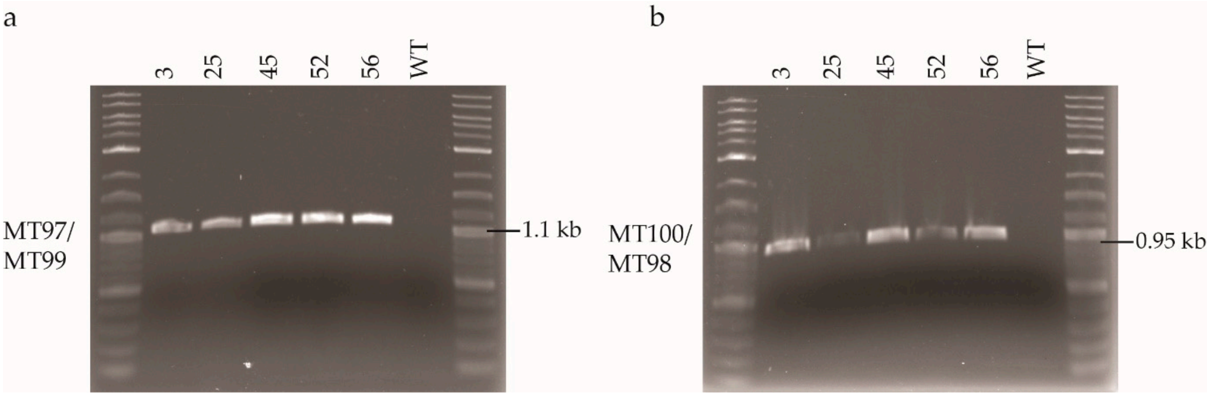

Figure S6. PCR screening to further characterise five *Dothistroma septosporum* CRISPR/Cas9 *Ds74283* transformants. (**a**) PCR across the junction of the 5′ flanking sequence and *nptII* with primer pair MT97/MT99. *Ds74283* mutants should have a product of 1.1 kb, while the wild-type (WT) fungus should not give any PCR product, since the primer MT99 binds to the promoter region of the *nptII* cassette. (**b**) PCR across the junction of *nptII* and the 3′ flanking sequence with primer pair MT100/MT98. *Ds74283* mutants should have a product of 0.95 kb, while the WT fungus should not give any PCR product, since the primer MT98 binds to the terminator region of the *nptII* cassette.

Table S1. Primers used in this study.

Name	Sequence 5′–3′	Purpose
HM95	GTCTCACGCGGCTCAGAGTCGAG	Amplification of sgRNA to be annealed and inserted into plasmid Cas9HygAMAccdB
HM96	AACCTCGACTCTGAGCCGCGTGA	
HM97	GTCACAAGAAGCAGCAGATAGGA	
HM98	AACTCCTATCTGCTGCTTCTTGT	
MT81	GTCGTTGTTGTAGGCAGAGACGA	
MT82	AACTCGTCTCTGCCTACAACAAC	
M139	TTTTCTCTTCCATTTACGC	Screening Cas9HygAMAccdB plasmid after insertion of gRNA
HM103	CGGTATGGGTACGCTCT	Amplification of *β-tubulin*
HM104	GAAATGGCACCTATCACAAG	
HM89	AACCCTACGCGTCTACCAG	Screening *AflR* transformants
HM90	GAAGAGGACTTCCGATCTTG	
HM183	GCAACTACTGGTGGTACG	
HM184	GCGCGAGATGTCCTTTC	
HM185	CTTGCTTGGCTTTGTCG	
HM186	GTGGAACGACATACCGG	
HM211	CTGACGGCACTGAGATC	
HM15	CGAAATTGCCGTCAACCAAGCTCT	Screening *AflR* transformants 82 and 158
HM16	TGTTTATCGGCACTTTGCATCGGC	
HM209	GAACATCGCCTCGCTCC	
HM210	GCCGCCTCCACCATTTG	
HM187	GTATGTCGTTCCACATCTAC	Amplification of *Ds140271*
HM188	CTTCAAGATGCCTTCTCC	
HM195	CATCCTAAGAGCTGCGG	Amplification of *AflJ*
HM196	GATGTCGCGGATCTCTC	
HM212	GTCGACCATGTTCGCC	Amplification of probe for Southern hybridization of *AflR*
HM213	GCAAGACTTCCTCCGAG	
MT85	GGGTTTTCCCAGTCACGACGTGCAGGCTGCGATACGAGA	Amplification of 5′ flanking sequence from *Ds74283*
MT86	GAACAACTGGCATGAATTCAAGCAACGAGGGCCAGAAAGA	
MT87	GGTAATCCTTCTTTCTAGATGCCTACAACAACACAACTGC	Amplification of 3′ flanking sequence from *Ds74283*
MT88	ACAATTTCACACAGGAAACAGCTGTACACGAGCTGCACGA	
MT89	GAATTCATGCCAGTTGTTCC	Amplification of *nptII* cassette from plasmid pII99
MT90	TCTAGAAAGAAGGATTACCTCTAAAC	
MT91	GCTGTTTCCTGTGTGAAATTG	Amplification of pAN-7 backbone for Gibson assembly of donor DNA
MT92	GTCGTGACTGGGAAAACCC	
MT95	ATGAAGTACCTTGCCCTCTTTCT	Screening *Ds74283* transformants
MT96	TCAGATGTATTGCGGAATGAGGT	
MT97	GAGGAATTGCTGATGAACAAGGTC	
MT98	AGAAGCTCGCTCATTATGTTTCCA	
MT99	TAGGCCGAATAACTTGCACAAATTG	
MT100	TTTACCCAGAATGCACAGGTACA	
MT101	GACAAACATCACCGCAAATG	Amplification of probe for Southern hybridization of *Ds74283* transformants
MT102	TGTACACGAGCTGCACGAAA	
